# Longitudinal Assessment of Health and Quality of Life of COVID-19 Patients Requiring Intensive Care—An Observational Study

**DOI:** 10.3390/jcm10235469

**Published:** 2021-11-23

**Authors:** Johanna Erber, Johannes R. Wießner, Gregor S. Zimmermann, Petra Barthel, Egon Burian, Fabian Lohöfer, Eimo Martens, Hrvoje Mijočević, Sebastian Rasch, Roland M. Schmid, Christoph D. Spinner, Rickmer Braren, Jochen Schneider, Tobias Lahmer

**Affiliations:** 1 Department of Internal Medicine II, University Hospital Rechts der Isar, School of Medicine, Technical University of Munich, 81675 Munich, Germany; johanna.erber@mri.tum.de (J.E.); JohannesRoman.Wiessner@mri.tum.de (J.R.W.); gregor.zimmermann@tum.de (G.S.Z.); Petra.Barthel@mri.tum.de (P.B.); Eimo.Martens@mri.tum.de (E.M.); sebastian.rasch@mri.tum.de (S.R.); RolandM.Schmid@mri.tum.de (R.M.S.); Christoph.spinner@mri.tum.de (C.D.S.); jochen.schneider@tum.de (J.S.); 2 Institute of Diagnostic and Interventional Radiology, University Hospital Rechts der ISAR, School of Medicine, Technical University of Munich, 81675 Munich, Germany; egon.burian@tum.de (E.B.); fabian.lohoefer@mri.tum.de (F.L.); Rickmer.Braren@mri.tum.de (R.B.); 3 Institute of Virology, School of Medicine, Technical University of Munich, 81675 Munich, Germany; hrvoje.mijocevic@tum.de

**Keywords:** COVID-19 sequelae, SARS-CoV-2, pulmonary function test, health-related quality of life, long-term health consequences

## Abstract

Long-term health consequences in survivors of severe COVID-19 remain unclear. Eighteen COVID-19 patients admitted to the intensive care unit at the University Hospital Rechts der Isar, Munich, Germany, between 14 March and 23 June 2020, were prospectively followed-up at a median of 36, 75.5, 122 and 222 days after discharge. The health-related quality of life (HrQoL) (36-item Short Form Health Survey and St. George’s Respiratory Questionnaire, SGRQ), cardiopulmonary function, laboratory parameters and chest imaging were assessed longitudinally. The HrQoL assessment revealed a reduced physical functioning, as well as increased SGRQ impact and symptoms scores that all improved over time but remained markedly impaired compared to the reference groups. The median radiological severity scores significantly declined; persistent abnormalities were found in 33.3% of the patients on follow-up. A reduced diffusion capacity was the most common abnormal pulmonary function parameter. The length of hospitalization correlated with role limitations due to physical problems, the SGRQ symptom and the impact score. In conclusion, in survivors of severe COVID-19, the pulmonary function and symptoms improve over time, but impairments in their physical function and diffusion capacity can persist over months. Longer follow-up studies with larger cohorts will be necessary to comprehensively characterize long-term sequelae upon severe COVID-19 and to identify patients at risk.

## 1. Introduction

While the global population of individuals recovering from severe acute respiratory syndrome coronavirus-2 (SARS-CoV-2) infection is growing as the virus spreads throughout continents, reports on persisting physical and mental health impairments are emerging, raising concerns about a potentially impeding chronic health issue.

Data from survivors of previous viral outbreaks, such as SARS and the Middle East respiratory syndrome coronavirus (MERS), show that pulmonary, as well as physical and mental function, may be impaired for months after discharge [1]. Whereas both the pulmonary function and radiological abnormalities seem to improve within the first years of recovery, residual pulmonary lesions and the persistent impairment of lung diffusion capacity have been described even 15 years after SARS infection [2]. 

The long-term health sequelae of SARS-CoV-2 infection remain to be elucidated. Follow-up studies performed in patients requiring intensive care due to severe COVID-19 are scarce. The few studies assessing lasting health sequelae in critical COVID-19 survivors report an impaired diffusion capacity and restrictive ventilatory defects on the pulmonary function test (PFT), persistent abnormalities on chest imaging and symptoms including fatigue and muscular weakness in the first months of convalescence [3,4,5]. 

In this study, 18 patients requiring ICU treatment for COVID-19 ARDS were longitudinally followed-up for seven months after discharge in order to perform a comprehensive health assessment comprising their health-related quality of life (HrQoL) and cardiopulmonary function, as well as chest imaging and laboratory parameters. 

## 2. Materials and Methods

### 2.1. Study Design and Participants

Eighteen out of 44 patients with PCR-confirmed SARS-CoV-2 infection admitted to one of the COVID-19 ICUs at the University Hospital Rechts der Isar, Technical University of Munich (TUM), between 14 March 2020 and 23 June 2020 were prospectively enrolled. Fifteen patients had died during hospitalization, one was lost to follow-up and all others were physically or mentally not able to attend follow-up appointments (FU). FUs were planned for 30 (FU1), 60 (FU2), 90 (FU3) and 180 (FU4) days upon discharge from the University Hospital Rechts der Isar, Technical University of Munich (TUM). Consultations were performed in our infectious disease clinic. Patients were interviewed by a physician and asked to self-complete the 36-item Short Form Health Survey (SF-36), as well as the St. George’s Respiratory Questionnaire (SGRQ), handwritten on paper. Vital parameters, a full blood count, creatinine, bilirubine, lactate dehydrogenase, C-reactive protein, procalcitonin, D-dimer, interleukin 6 and troponine T were assessed. Anti-SARS-CoV-2-IgG and -IgM antibody levels were measured using a paramagnetic particle chemiluminescent immunoassay (CLIA) on an iFlash 1800 immunoassay analyser (Shenzhen Yhlo Biotech Co., Shenzhen, China). An electrocardiogram (ECG) and PFT were performed at each visit. A transthoracic echochardiography (TTE) and computer tomography of the chest (CT) were planned for FU3 and facultatively FU4. Demographic, clinical and laboratory data of the acute phase (defined as the time between symptom onset and hospital discharge) were retrospectively compiled by chart review. The disease severity was characterized using the WHO clinical progression scale [6]. 

### 2.2. Cardiopulmonary Diagnostic

TTEs were performed by specialist cardiologists, and the ECG and the PFT were performed by trained staff of the Department of Internal Medicine I (Cardiology, Angiology and Pneumology). The PFT was performed following the European Respiratory Society Recommendations [7,8] and included a combined spirometry with whole-body plethysmography (MasterScreen Body, Jaeger, Wuerzburg, Germany); if applicable, a measurement of diffusing capacity of carbon monoxide (DLCO) was added (MS-PFT, Jaeger, Wuerzburg, Germany). Pulmonary parameters included the vital capacity (VC), forced vital capacity (FVC), forced expiratory volume at the first second of maximal expiration (FEV1), FEV1/FVC ratio and total lung capacity (TLC). Further, the DLCO, the alveolar volume (VA) and the carbon monoxide transfer rate (KCO) were measured. The DLCO and KCO were adjusted for the hemoglobin level. The results are expressed as the percentage of the predicted values by Zapletal (TLC, VC) and Quanjer (FEV1, FEV1/FVC ratio) [9,10]. Values ≤ 80% of the predicted value were defined as abnormal. Restrictive lung disease was defined if the TLC was ≤80% of the predicted value with concurrent normal FEV1/FVC% ratio (>80%). 

### 2.3. CT Image Acquisition and Analysis

Chest CTs were performed in the supine position in end-inspiration. All patients were examined with the same 256-row multidetector computed tomography (MDCT) scanner (iCT, Philips Healthcare, Best, The Netherlands). The chest CTs were evaluated by two radiologists (5 and 9 years of experience) blinded to the clinical data, and severity scores ranging from 0 to 5 were determined as previously described [11]. 

### 2.4. Questionnaires

Scoring of the SF-36 was performed as recommended by RAND Health Care (https://www.rand.org/health-care/surveys_tools/mos/36-item-short-form/scoring.html, accessed on 31 August 2021): All 36 items are scored on a 0 to 100 range, with higher scores representing a more favorable subjective health state. Subsequently, items relating to one of the eight distinct dimensions (physical functioning, social functioning, role limitations due to physical problems, role limitations due to emotional problems, mental health, vitality, pain and general health) are averaged. The values reported for healthy men aged 45–54 were considered as reference [12]. Scores of the SGRQ for the two subscales ‘symptoms’ and ‘impacts’ were calculated using the Excel scoring calculator provided by the developer (P. W. Jones, St. George’s Hospital Medical School, London, UK). The activity score could not be evaluated, as the respective part of the questionnaire had not been printed correctly. The scores of all subscales range from zero (no impairment) to 100 (maximum impairment). A change in at least 4 points has been described as clinically relevant [13]. The mean SGRQ scores for the age group 50–59 as reported by Ferrer et al. were used as reference [14]. All questionnaire data are presented as mean with SD. 

### 2.5. Statistical Analysis

Statistical analyses were conducted using Microsoft Excel (V16.51) and GraphPad Prism 9.0. Unless otherwise stated, the Anderson–Darling test was applied to test for normal distribution. Normally distributed data are presented as mean with standard deviation (±SD), and data not fitting normal distribution are presented as median with interquartile range (IQR). Correlation was tested using nonparametric Spearman correlation. Significance between the CT scans from the acute phase and the FU were tested using the Wilcoxon matched-pairs signed rank test, and significance between FU visits for all other assessments was tested by one-way-ANOVA with Dunnett’s multiple comparison test and is indicated by asterisks (* = *p* < 0.05; ** = *p* < 0.01; *** = *p* < 0.001). Missing data due to failed attendance or inability to complete a test are depicted in tables and figures when appropriate, and missing data were not imputed. Graphs were plotted using R studio (1.4.1106) and GraphPad Prism 9.0 and assembled using Adobe Illustrator 2021. 

## 3. Results

All 18 eligible patients had been admitted to the ICU due to respiratory failure (pO_2_/FiO_2_ ratio on admission 183.5, IQR, 71–272 mmHg requiring oxygen support and meeting the Berlin criteria for ARDS [15] (median WHO clinical progression scale 8, IQR, 5–9). High flow nasal cannula (HFNC) treatment was applied in one patient before intubation, and 13 patients (72.2%) required mechanical ventilation with a median length of 9 days (IQR, 2–63 days). Prone positioning was applied in 6/18 patients (33.3%), and extracorporeal membrane oxygenation therapy was required in one patient. Four patients (22.2%) received remdesivir treatment, two patients received convalescent plasma (11.1%) and one patient received dexamethasone treatment. The median length of the stay in ICU was 10 days (IQR, 1–71 days) and the median duration of hospitalization was 21.5 days (IQR, 8–71 days). The detailed clinical characteristics of the participants are shown in Table 1. 

The follow-up study was conducted between 18 May 2020 and 1 February 2021. The median time points between hospital discharge and the FUs were 36 (FU1, IQR, 31–65 days), 75.5 (FU2, IQR, 62–107 days), 122 (FU3, IQR, 94–147 days) and 222 days (FU4, IQR, 194–252 days, see Table 2 for detailed data from the follow-up study). 

**Figure 1:**
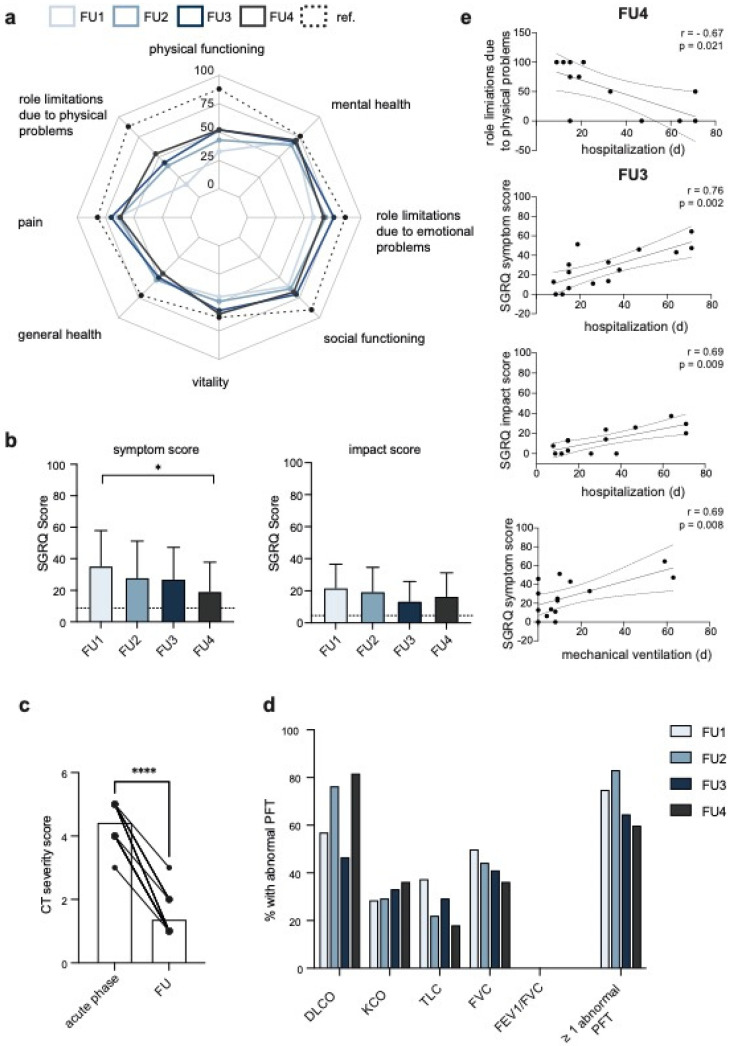
Follow-up assessments of HrQoL, pulmonary function and radiological CT severity scores in critical COVID-19 survivors. (**a**) The radar plot shows the SF-36 scores of eight dimensions for all FUs and reference values (healthy men aged 45–54) in dashed lines. A score of 100 represents the best subjective health state. (**b**) Boxplots show the mean (±SD) SGRQ symptom (left panel) and impact score (right panel) of all follow-up assessments (FU). The dashed lines indicate mean reference scores for the age group 50–59. (**c**) The CT severity scores from chest CTs performed at the time of admission (acute phase) and during follow-up (119 ± 15.3 days upon discharge) are plotted, and the lines connect the scores of each individual patient. (**d**) The percentage of patients with abnormal PFTs (≤80% of the predicted value) is presented. (**e**) The scatter plots show the Spearman correlations with a 95% confidence interval for the FU3 or FU4. The Spearman r and *p*-values are annotated. See Figure S3 for all FUs. Significance was tested by the one-way-ANOVA with Dunnett’s multiple comparison test (**b**) and by the Wilcoxon matched-pairs signed rank test (**c**), and is indicated by asterisk (* = *p* < 0.05; **** = *p* < 0.0001). Non-significant levels are not labelled. CT, computed tomography; d, days; DLCO, lung diffusion capacity for CO; FEV1, forced expiratory volume at the first second of maximal expiration; FVC, forced vital capacity; FU, follow-up appointment; KCO, carbon monoxide transfer rate; PFT, pulmonary function test; ref., reference; SGRQ, St. George’s Respiratory Questionnaire; TLC, total lung capacity.

Two validated instruments were used to longitudinally evaluate the health-related quality of life (HrQoL). As assessed by the SF-36, the strongest impairments at the initial visit (FU1) were reported for the dimensions ‘role limitation due to physical problems’ (16.1 ± 31.9), followed by ‘physical functioning’ (33.3 ± 31.7). Compared with healthy male individuals of a similar age, impairments in the dimensions ‘social functioning’ (60.7 ± 27.2), ‘role limitations due to emotional problems’ (58.3 ± 47.4) and ‘general health’ (60.7 ± 27.2) were striking. (Figure 1a, Table 2). While the impairment in the dimension ‘general health’ remained impaired during follow-up, the role limitations due to physical problems, as well as the impairment in physical functioning, significantly decreased, but remained markedly lower compared to the reference group (see Table 2 for *p*-values). 

The SGRQ is a standardized 50-item questionnaire that is widely used in patients with respiratory disorders. Compared with the reference group, both the SGRQ symptom and impact score were markedly increased at all follow-up visits (symptom score: mean 35.4, 28.1, 27.1, 19.3 at FU1–4, respectively, reference 8.7; impact score: mean 21.9, 19.5, 13.4, 16.7, reference 4.6). The SGRQ symptom score significantly improved by 16 points from FU1 to FU4 (*p* = 0.01), whereas the impact score improved by five points from FU 1 to FU4 (Figure 1b, Table 2). The most common symptoms reported at the FU4 were paraesthesia (8/15, 53.3%), fatigue (5/15, 33.3%) and exertional dyspnoea (4/15, 26.7%). Both dysgeusia and olfactory dysfunction persisted in 20% (3/15) of the patients. Three of the patients required hospitalization during follow-up. 

The radiological severity scores were assessed and compared between chest CTs performed at the time of admission (acute phase) and during follow up (119 ± 15.3 days upon discharge). The median severity score of the initial scan was 4.5 (IQR, 3–5), which significantly declined to a median score of 1 (IQR, 1–3) on the FU3 (Figure 1c, *p* < 0.0001). 33.3% (6/18) of the patients still had abnormal findings on the follow-up. The predominant radiological appearance in most of the patients was disseminated ground glass opacities mixed with partial consolidation (Figure S1).

An abnormal PFT (≥one parameter ≤80% of the predicted value) was assessed in 75.0, 83.3, 64.7 and 64.3% of all patients performing a PFT at the FU1, FU2, FU3 and FU4, respectively (Figure 1d and Figure S2). The most common pulmonary function abnormality at all follow-up visits was an abnormal reduction in the DLCO (FU1: 57.1%, FU2: 76.5%, FU3: 46.7%, FU4: 81.8%), followed by a reduction in the FVC (Figure 1d and Figure S2, Table 2) and TLC (Figure 1d and Figure S2, Table 2). 

Whereas the frequency of abnormally reduced DLCO measurements was higher in the patients evaluated at the FU4 compared to the FU1, the amount of abnormal TLC and FVC measurements markedly declined from the FU1 to the FU4. An increase in the DLCO, FVC and TLC over time (>10% between the first and last PFT performed) was observed in 47.1% (8/17), 33.3% (6/18) and 50% (9/18) of the patients, respectively (Figure S2). 

Correlating the most abnormal PFTs, the HrQoL parameters, the CT severity scoring and length of mechanical ventilation, as well as the ICU stay and hospitalization, revealed an association of the length of hospitalization with the role limitations due to physical problems (FU2: r = −0.6, *p* = 0.016; FU4: r = −0.67, *p* = 0.021), as well as the SGRQ symptom score (FU3: r = 0.76, *p* = 0.002) and the SGRQ impact score (FU3: r = 0.69, *p* = 0.009) (Figure 1e and Figure S3A–C). Further, we observed a correlation of the length of mechanical ventilation and the SGRQ symptom score (FU3: r = 0.69, *p* = 0.008) (Figure 1 and Figure S3D). Moreover, the impact score was higher in patients with a decreased FVC at the FU1 (r = −0.63, *p* = 0.03) and the CT severity scoring on the follow-up negatively correlated with the FVC at the FU1 (r = −0.54, *p* = 0.03) (Figure S3E,F). 

The body mass indices did not significantly change over time compared to the acute phase (Figure S4A, Table 2). The vital parameters remained stable over the FUs, apart from a significant decrease (*p* = 0.012) in the mean diastolic blood pressure at FU4 compared to FU1 (Figure S4B–D, Table 2). Anti-SARS-CoV-2-IgG was positive in 93.8% (15/16) of patients at the time of hospitalization, and remained positive in 88.9% (16/18) of patients at the FU1–3. At the FU4, four patients were already seronegative, and the anti-SARS-CoV-2-IgG levels had significantly decreased compared with the acute phase (*p* = 0.022). Anti-SARS-CoV-2 IgM was positive in 27.8% of patients at the FU 1 (5/18), and all patients were seronegative at the FU3 (15/15) (Figure S5A). Both increased and decreased platelet levels were observed during hospitalization, which had been normalized at the time of follow-up (Figure S5B). The serum levels of creatinine, bilirubin, LDH and the inflammatory parameters CRP, PCT, IL-6 and leukocytes were elevated during the acute phase of the infection, but returned to normal values or baseline levels in the case of pre-existing chronic kidney disease and remained stable during the follow-up (Figure S5C–K).

ECG diagnostics showed an Osborne wave in one patient at admission and during the follow-up, possibly in the context of a diffuse early repolarization syndrome. The same patient developed a first-degree atrioventricular block during the follow-up. TTE was available in 14 patients: hemodynamically irrelevant pericardial effusion was found in two patients. One patient had signs for pulmonary arterial hypertension following surgical aortic valve replacement; other than that, no relevant abnormalities were found (data not shown). In the acute phase, bilateral central pulmonary emboli were diagnosed in one patient, and venous thromboembolism was diagnosed in two patients. No thrombotic event was noted during the follow-up.

## 4. Discussion

To our best knowledge, this is the first study longitudinally monitoring health consequences of patients requiring intensive care for COVID-19 ARDS 9 months from symptom onset.

The SF-36 and SGRQ questionnaires revealed significant impairments in several dimensions of the HrQoL. Whereas most of the SF-36 and both SGRQ scores improved during convalescence, the subjective perception of the general health status deteriorated over time. Similar findings have been reported by Hui et al. in a 12-month follow-up study of SARS survivors (*n* = 97, including 31 ICU patients) [16]. In our cohort of ICU survivors, the physical functioning and role limitation due to physical problems improved during the first months of convalescence, but remained remarkably low seven months after discharge. Further, the length of hospitalization appeared to correlate with persistent role limitations due to physical problems and both the SGRQ symptom and impact score. In line with this, Huang et al. report muscle weakness as one of the most common persisting symptoms six months from symptom onset in a cohort of 1733 hospitalized COVID-19 patients, which was increased in patients with more severe disease, suggesting that physical impairment is long-lasting in patients requiring critical care for SARS-CoV-2-related ARDS and is potentially attributable to limited mobility and physical therapy in the context of isolation. In addition to muscle weakness, the majority of the patients reported persistent paraesthesia, which is suggestive of ICU-acquired weakness (ICUAW), which comprises critical illness myopathy (CIM) and critical illness polyneuropathy (CIP). CIM and CIP were described in 6.3 and 7.6% of 1264 critically ill COVID-19 patients of a multi-center European cohort study, respectively [17]. However, ICUAW is generally common in critically ill patients [18], making it difficult to determine whether the occurrence is a non-specific manifestation of critical illness or is related to SARS-CoV-2. Intriguingly, mental health appeared to be only mildly affected in our cohort, which differs from data shown by Huang et al. and data from SARS and MERS survivors that report that mental health problems, such as stress, anxiety and depression, are prevalent in up to one third of the patients followed-up for six months. Whether socioeconomic factors or rehabilitation measures contribute to this difference remains to be investigated.

The radiological severity grading had significantly improved after four months; however, persistent abnormalities were found in more than one third of the patients, which is in line with previous studies reporting the persistence of primarily ground glass opacity in COVID-19 survivors [3,4,5].

A compromised respiratory function, mostly restrictive in nature, appears to be the main issue in survivors of coronavirus infections [1,19,20]. Similar to other COVID-19 follow-up studies, an impaired diffusion capacity was the most common pulmonary function parameter in our follow-up study [3,21]. While Huang et al. found an abnormal DLCO in 56% of the patients requiring HFNC, non-invasive or invasive ventilation six months after symptom onset, here, the DLCO was impaired in 46.7% of the patients presenting at the FU3 (5 months after symptom onset), and in more than 80% at the FU4 (9 months from symptom onset). A restrictive defect was observed in 37.5% of the patients at the initial FU, and remained in 18.2% of the patients presenting at the FU4. Although pre-existing COPD and asthma were reported in two and one patients, respectively, we, along with others, did not find any obstructive ventilatory defect in COVID-19 survivors, even if mechanical ventilation was applied [5].

We note that persistent pulmonary fibrosis on radiological examination has been described up to fifteen years after SARS infection, and the pulmonary function of patients with persistent radiological abnormalities was worse compared to patients without abnormal findings on CT [2,22]. Preliminary data from a shorter COVID-19 follow-up study suggest that the disease severity correlates with diffusion impairment and higher CT scores [3]. Larger long-term follow-up studies will be required to investigate whether the persistent radiological and pulmonary function abnormalities completely resolve over time.

In this cohort, the laboratory and cardiological examination did not indicate any severe sequelae or unprecedented disorders following severe COVID-19. This contrasts with other studies, which have emphasized persisting radiological and functional signs of cardiac damage on cardiac MRI and transthoracic echocardiography scans [23,24]. However, elevated cardiac markers were noted in nine patients during the acute phase, possibly foreshadowing cardiac long-term sequelae.

The declining anti-SARS-CoV2 antibody titers should serve as a reminder to recommend COVID-19 survivors getting vaccinated in order to prevent SARS-CoV2 re-infection.

The strengths of this study were the prospective design and the longitudinal and comprehensive assessment of mental and physical health. However, the study was monocentric and the cohort was limited in size. Given the uncontrolled nature, we cannot exclude that some of the observed effects are not related to COVID-19, but result from the critical care, including invasive ventilation.

The enrolment might have been biased, as patients were only enrolled if physically and mentally well enough to attend follow-up visits. Thus, our data might overestimate the health status of patients surviving critical COVID-19. At the same time, missing data points resulted from patients failing to complete a distinct test or attend the appointment and limited correlations and multivariable analyses. We hypothesize that patients suffering from persistent symptoms are more likely to keep the study-related follow-up appointments. However, we cannot exclude that non-attendance might be explained by severe physical impairment or death. This study only comprises patients treated early in the pandemic, explaining the underrepresentation of non-invasive respiratory support strategies and application of dexamethasone. The long-term effects of treatment approaches, such as antiviral drugs, dexamethasone or monoclonal antibodies, remain unknown. Our data are limited, as TTE, pulmonary function tests or SF-36 and SGRQ scores from pre-admission or the acute phase were not available. Given that several patients enrolled in this study had significant comorbidities, including COPD, chronic kidney disease or cancer, their baseline SF-36 or SGRQ scores might have been altered, leading to a bias when comparing COVID-19 patients with healthy reference groups. Moreover, ≤80% of the predicted value was used as the threshold for abnormal pulmonary function, although the lower limit of normal would have been statistically more adequate.

## 5. Conclusions

We and others report that, even in patients requiring intensive care for COVID-19 ARDS, the radiological abnormalities, pulmonary function and symptoms improve over time. However, impairments, particularly in their physical function and the DLCO, remain over months in a substantial number of patients surviving critical COVID-19. Longer multicentric follow-up studies with larger cohorts will be required in order to grasp the full spectrum and extent of health sequelae, investigate the underlying pathophysiological mechanisms and characterize biomarkers in order to identify patients that are at an increased risk for long-term sequelae and therefore require closer monitoring or intensified rehabilitation upon critical COVID-19.

## Figures and Tables

**Table 1:** Demographics, baseline characteristics and clinical course.

Baseline Characteristics	Mean (SD) or Median (Range)	n/N (%)
Age (years)	54 (12.3)	
Female		4/18 (22.2)
Male		14/18 (77.8)
BMI (kg/m^2^)	27.8 (5.3)	
Current smoker		1/18 (5.6)
Former smoker		6/18 (33.3)
**Comorbidities**		
Hypertension		8/18 (44.4)
Diabetes mellitus		4/18 (22.2)
Adipositas		4/18 (22.2)
Coronary heart disease		1/18 (5.6)
Congestive heart failure		0/18 (0.0)
COPD		2/18 (11.1)
Asthma bronchiale		1/18 (5.6)
Chronic kidney disease		4/18 (22.2)
Chronic liver disease		2/18 (11.1)
Cancer		3/18 (16.7)
HIV		1/18 (5.6)
Immunosuppression		3/18 (16.7)
None		5/18 (27.8)
**Admission details**		
SOFA score at admission to ICU	3.5 (1–16)	
Apache II score at admission to ICU	15.5 (8.3)	
pO_2_/FiO_2_ at admission	183.5 (71–272)	
Length of stay on ICU (days)	10 (1–71)	
Length of stay in hospital (days)	21.5 (8–71)	
**Critical care**		
Oxygen		18/18 (100)
NIV		0/0 (0)
HFNC		1/18 (5.6)
Mechanical ventilation		13/18 (72.2)
Length of mechanical ventilation (days)	9 (2–63)	
Proning		6/18 (33.3)
ECMO therapy		1/18 (5.6)
Renal replacement therapy		4/18 (22.2)
WHO clinical progression score	8 (5–9)	
**COVID-19 directed therapy**		
Remdesivir		4/18 (22.2)
Dexamethason		1/18 (5.6)
Convalescent plasma		2/18 (11.1)

Data are presented as mean (standard deviation) if the Anderson–Darling normality test was passed; alternatively, the median (range) is reported. For nominal data, n/N (%) is reported, where N is the total number of participants with available data. BMI, body mass index; COPD, chronic obstructive pulmonary disease; ECMO, extracorporeal membrane oxygenation; HFNC, high flow nasal cannula; HIV, human immunodeficiency virus; ICU, intensive care unit; NIV, non-invasive ventilation; SD, standard deviation; SOFA, sequential organ failure assessment; WHO, World Health Organization.

**Table 2:** Data from health assessments at four follow-up appointments.

	FU 1	FU 2	FU3	FU4
Follow-Up Details	Mean (SD) or Median (Range) [*p*-Value, If Significant]			
Median time from hospital discharge (d)	36 (31–65)	75.5 (62–107)	122 (94–147)	222 (194–252)
Median time from symptom onset (d)	73.5 (54–124)	109.5 (89–186)	159.5 (125–214)	263 (216–312)
Failure to attempt FU (number)	0	0	0	1
**SF-36**				
Role limitations due to physical problems Missing values	16.1 (31.9) 4	39.1 (37.6) 3	43.3 (46.7) [0.029] 3	54.2 (43.7) [0.002] 6
Physical functioning Missing values	33.3 (31.7) 4	43.1 (31.9) 0	51.7 (37.2) [0.04] 3	51.9 (36.0) 5
Mental health Missing values	69.5 (17.6) 4	65.2 (23.2) 0	69.3 (18.1) 3	70.6 (17.7) 5
Role limitations due to emotional problems Missing values	58.3 (47.4) 6	68.5 (40.4) 0	75.6 (42.7) 3	66.7 (40.8) 5
Social functioning Missing values	60.7 (27.2) 4	63.9 (29.7) 0	70.8 (28.6) 3	68.3 (30.5) 5
Vitality Missing values	45.0 (11.9)	49.4 (20.5)	56.7 (22.5)	59.6 (20) [0.023]
General health Missing values	51.8 (13.5) 4	52.6 (10.6) 1	49.6 (11.7) 4	45.2 (11.1) 6
Pain Missing values	64.6 (22.1) 4	62.8 (28.1) 0	69.5 (30.4) 3	61.9 (26.7) 5
**SGRQ**				
Symptom score Missing values	35.4 (22.5) 4	28.1 (23.1) 1	27.1 (20) 3	19.3 (18.5) [0.01] 5
Impact scoreMissing values	21.9 (14.7) 5	19.5 (15.1) 3	13.4 (12.4) 4	16.7 (14.6) 5
**Vital signs**				
Systolic blood pressure (mmHg)	137.7 (20.8)	137.2 (21.4)	137.4 (15.7)	134.0 (17.1)
Diastolic blood pressure (mmHg)	90.2 (8.5)	89.7 (9.3)	90.4 (9.2)	76.5 (18.9)
Heart rate (beats per minute)	78.8 (12.9)	75.0 (10.8)	74.8 (11.4)	80.0 (14.4)
Peripheral oxygen saturation (%)	96.29 (4.12)	97.22 (1.31)	97.06 (1.76)	98.33 (1.63)
BMI (kg/m^2^) Missing values	26.1 (19.1–44.9) 0	26.9 (18.4–42.3) 0	27.1 (19.1–49.2) 1	27.6 (24.7–47.9) 5
**Pulmonary Function Test**	**Mean (SD) [n/N with Abnormal PFT]**			
DLCO	70.6 (20.3) [8/14]	69.3 (18.8) [13/17]	77.5 (19.3) [7/15]	66.5 (14.8) [9/11]
FVC	79.7 (12.1) [8/16]	81.7 (11.5) [8/18]	81.3 (10.7) [7/17]	83.5 (12.4) [4/11]
TLC	84.7 (13.0) [6/16]	90.3 (13.3) [4/18]	88.4 (12.9) [5/17]	97.4 (12.7) [2/11]
KCO	90.0 (20.8) [4/14]	89.6 (18.8) [5/17]	91.3 (19.8) [5/15]	85.0 (17.3) [4/11]
VC	81.9 (13.7) [8/16]	84.9 (12.0) [6/18]	83.5 (10.5) [5/17]	86.8 (11.8) [4/11]
FEV1	83.6 (10.7) [7/16]	86.4 (9.8) [6/18]	85.2 (11.2) [8/17]	86.0 (14.4) [5/11]
FEV1/FVC ratio in %	103.6 (5.9) [0/14]	104.8 (8.2) [0/17]	103.4 (5.1) [0/15]	102.7 (8.3) [0/11]
VA	78.6 (13.6) [7/14]	79.4 (13.5) [10/17]	85.4 (10.4) [5/15]	80.3 (9.6) [5/11]

Data are presented as mean (standard deviation) if the Anderson–Darling normality test was passed; alternatively, the median (range) is reported. For nominal data, n/N is reported, where N is the total number of participants with available data. BMI, body mass index; d, days; DLCO, lung diffusion capacity for CO; FEV1, forced expiratory volume at the first second of maximal expiration; FU, follow-up appointment; FVC, forced vital capacity; KCO, carbon monoxide transfer rate; PFT, pulmonary function test; SF-36, 36-item Short Form Health Survey; SGRQ, St. George’s Respiratory Questionnaire; TLC, total lung capacity; VA, alveolar volume; VC, vital capacity.

## Data Availability

All raw data is provided in Table S1.

## Supplementary Materials

The following are available online at https://www.mdpi.com/article/10.3390/jcm10235469/s1, Figure S1: Follow-up assessments of HrQoL, pulmonary function and radiological CT severity scores in critical COVID-19 survivors, Figure S2: Longitudinal assessment of the pulmonary function, Figure S3: Selected Spearman correlations of clinical data, pulmonary function and HrQoL, Figure S4: Longitudinal assessment of vital parameters and body mass index, Figure S5: Longitudinal assessment of laboratory parameters and anti-SARS-CoV2 IgG and IgM. Table S1: Raw data of all patients from the acute phase and follow-up visits.

## References

[1] Ahmed H., Patel K., Greenwood D.C., Halpin S., Lewthwaite P., Salawu A., Eyre L., Breen A., O’Connor R., Jones A. (2020). Long-term clinical outcomes in survivors of severe acute respiratory syndrome and Middle East respiratory syndrome coronavirus outbreaks after hospitalisation or ICU admission: A systematic review and meta-analysis. J. Rehabil. Med..

[2] Zhang P., Li J., Liu H., Han N., Ju J., Kou Y., Chen L., Jiang M., Pan F., Zheng Y. (2020). Long-term bone and lung consequences associated with hospital-acquired severe acute respiratory syndrome: A 15-year follow-up from a prospective cohort study. Bone Res..

[3] Huang C., Huang L., Wang Y., Li X., Ren L., Gu X., Kang L., Guo L., Liu M., Zhou X. (2021). 6-month consequences of COVID-19 in patients discharged from hospital: A cohort study. Lancet.

[4] Stylemans D., Smet J., Hanon S., Schuermans D., Ilsen B., Vandemeulebroucke J., Vanderhelst E., Verbanck S. (2021). Evolution of lung function and chest CT 6 months after COVID-19 pneumonia: Real-life data from a Belgian University Hospital. Respir. Med..

[5] Orzes N., Pini L., Levi G., Uccelli S., Cettolo F., Tantucci C. (2021). A prospective evaluation of lung function at three and six months in patients with previous SARS-CoV-2 pneumonia. Respir. Med..

[6] Marshall J.C., Murthy S., Diaz J., Adhikari N.K., Angus D.C., Arabi Y.M., Baillie K., Bauer M., Berry S., Blackwood B. (2020). A minimal common outcome measure set for COVID-19 clinical research. Lancet Infect. Dis..

[7] Wanger J., Clausen J.L., Coates A., Pedersen O.F., Brusasco V., Burgos F., Casaburi R., Crapo R., Enright P., van der Grinten C.P. (2005). Standardisation of the measurement of lung volumes. Eur. Respir. J..

[8] Coates A.L., Peslin R., Rodenstein D., Stocks J. (1997). Measurement of lung volumes by plethysmography. Eur. Respir. J..

[9] Zapletal A., Paul T., Samánek M. (1977). Significance of contemporary methods of lung function testing for the detection of airway obstruction in children and adolescents (author’s transl). Z. Erkrank. Atm..

[10] Quanjer P.H., Stanojevic S., Cole T.J., Baur X., Hall G.L., Culver B.H., Enright P.L., Hankinson J.L., Ip M.S., Zheng J. (2012). Multi-ethnic reference values for spirometry for the 3–95-yr age range: The global lung function 2012 equations. Eur. Respir. J..

[11] Burian E., Jungmann F., Kaissis G.A., Lohöfer F.K., Spinner C.D., Lahmer T., Treiber M., Dommasch M., Schneider G., Geisler F. (2020). Intensive Care Risk Estimation in COVID-19 Pneumonia Based on Clinical and Imaging Parameters: Experiences from the Munich Cohort. J. Clin. Med..

[12] Jenkinson C., Coulter A., Wright L. (1993). Short form 36 (SF36) health survey questionnaire: Normative data for adults of working age. Br. Med. J..

[13] Meguro M., Barley E.A., Spencer S., Jones P.W. (2007). Development and Validation of an Improved, COPD-Specific Version of the St. George Respiratory Questionnaire. Chest.

[14] Ferrer M., Villasante C., Alonso J., Sobradillo V., Gabriel R., Vilagut G., Masa J.F., Viejo J.L., Jiménez-Ruiz C.A., Miravitlles M. (2002). Interpretation of quality of life scores from the St George’s Respiratory Questionnaire. Eur. Respir. J..

[15] Ranieri V.M., Rubenfeld G.D., Thompson B.T., Ferguson N.D., Caldwell E., Fan E., Camporota L., Slutsky A.S. (2012). Acute respiratory distress syndrome: The Berlin Definition. JAMA.

[16] Hui D.S., Wong K.T., Ko F.W., Tam L.S., Chan D.P., Woo J., Sung J.J. (2005). The 1-year impact of severe acute respiratory syndrome on pulmonary function, exercise capacity, and quality of life in a cohort of survivors. Chest.

[17] Kleineberg N.N., Knauss S., Gülke E., Pinnschmidt H.O., Jakob C.E.M., Lingor P., Hellwig K., Berthele A., Höglinger G., Fink G.R. (2021). Neurological symptoms and complications in predominantly hospitalized COVID-19 patients: Results of the European multinational Lean European Open Survey on SARS-Infected Patients (LEOSS). Eur. J. Neurol..

[18] Bolton C.F., Young G.B., Zochodne D.W. (1993). The neurological complications of sepsis. Ann. Neurol..

[19] Hui D.S., Joynt G.M., Wong K.T., Gomersall C.D., Li T.S., Antonio G., Ko F.W., Chan M.C., Chan D.P., Tong M.W. (2005). Impact of severe acute respiratory syndrome (SARS) on pulmonary function, functional capacity and quality of life in a cohort of survivors. Thorax.

[20] Ngai J.C., Ko F.W., Ng S.S., To K.W., Tong M., Hui D.S. (2010). The long-term impact of severe acute respiratory syndrome on pulmonary function, exercise capacity and health status. Respirology.

[21] Ekbom E., Frithiof R., Öi E., Larson I.M., Lipcsey M., Rubertsson S., Wallin E., Janson C., Hultström M., Malinovschi A. (2021). Impaired diffusing capacity for carbon monoxide is common in critically ill COVID-19 patients at four months post-discharge. Respir. Med..

[22] Wu X., Dong D., Ma D. (2016). Thin-Section Computed Tomography Manifestations During Convalescence and Long-Term Follow-Up of Patients with Severe Acute Respiratory Syndrome (SARS). Med. Sci. Monit..

[23] Bieber S., Kraechan A., Hellmuth J.C., Muenchhoff M., Scherer C., Schroeder I., Irlbeck M., Kaeaeb S., Massberg S., Hausleiter J. (2021). Left and right ventricular dysfunction in patients with COVID-19-associated myocardial injury. Infection.

[24] Weckbach L.T., Curta A., Bieber S., Kraechan A., Brado J., Hellmuth J.C., Muenchhoff M., Scherer C., Schroeder I., Irlbeck M. (2021). Myocardial Inflammation and Dysfunction in COVID-19-Associated Myocardial Injury. Circ. Cardiovasc. Imaging.

