# Supplementary material for: Longitudinal Assessment of Health and Quality of Life of COVID-19 Patients Requiring Intensive Care—An Observational Study
Source: J Clin Med. 2021 Nov 23;10(23):5469. doi: 10.3390/jcm10235469 (PMC8658191; doi:10.3390/jcm10235469)
Supplement: Supplementary file 1 [file jcm-10-05469-s001.zip › Supplementary files_MDPI.pdf]

Longitudinal Assessment of Health and Quality of Life of COVID-19 Patients Requiring Intensive Care—An Observational Study

Johanna Erber [1], Johannes R. Wießner [1], Gregor S. Zimmermann [1], Petra Barthel [1], Egon Burian [2], Fabian Lohöfer [2], Eimo Martens [1], Hrvoje Mijočević [3], Sebastian Rasch [1], Roland M. Schmid [1], Christoph D. Spinner [1], Rickmer Braren [2], Jochen Schneider [1] and Tobias Lahmer [1,*]

Figure S1 2

Figure S2 3

Figure S3 4

Figure S4 5

Figure S5 6

Figure S1

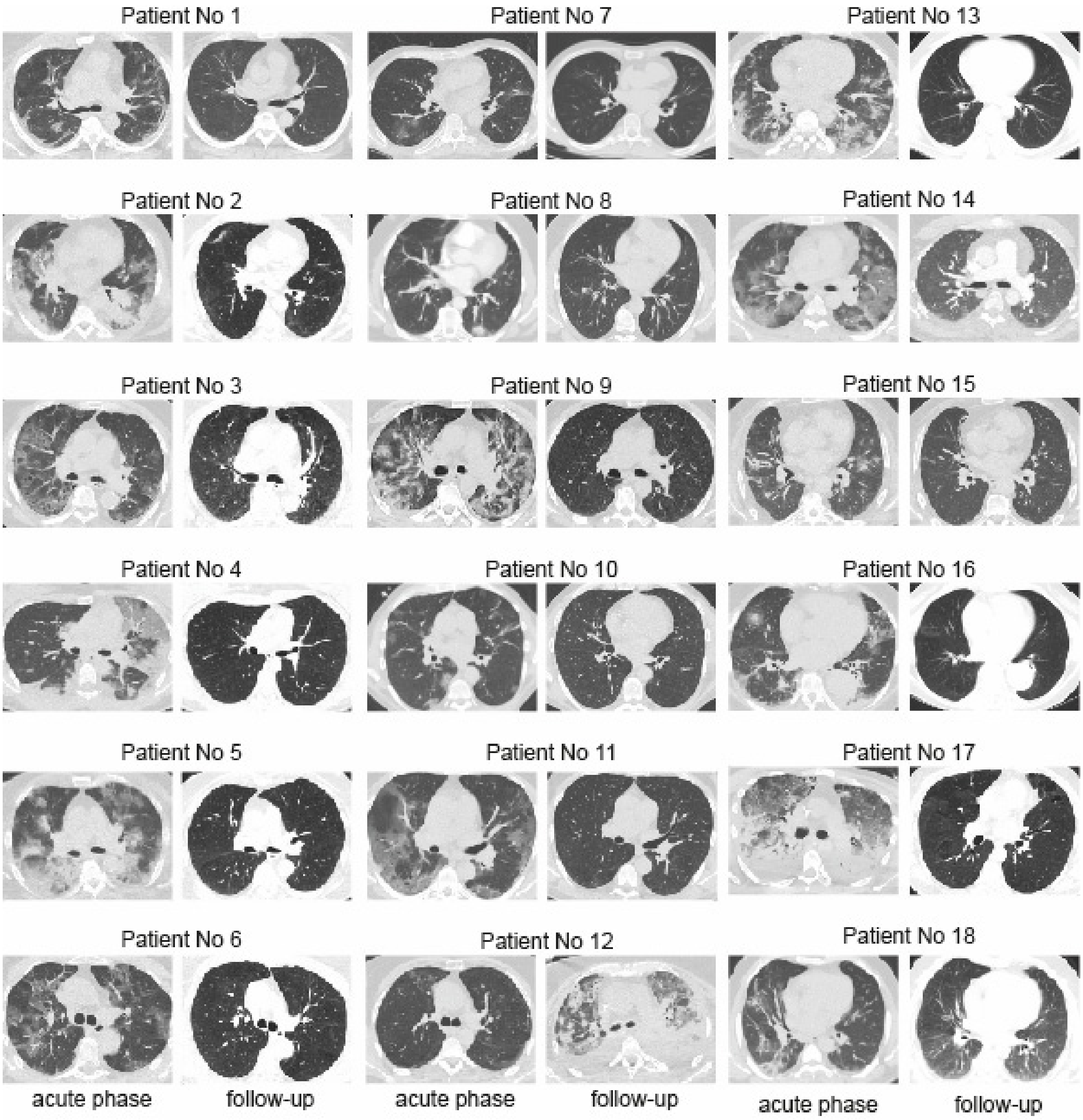

Figure S1. Follow-up assessments of HrQoL, pulmonary function and radiological CT severity scores in critical COVID-19 survivors Representative CT images from the acute phase (left image) and follow-up (right image) are depicted for all 18 patients. The respective severity scores are presented in Figure 1c.

Figure S2

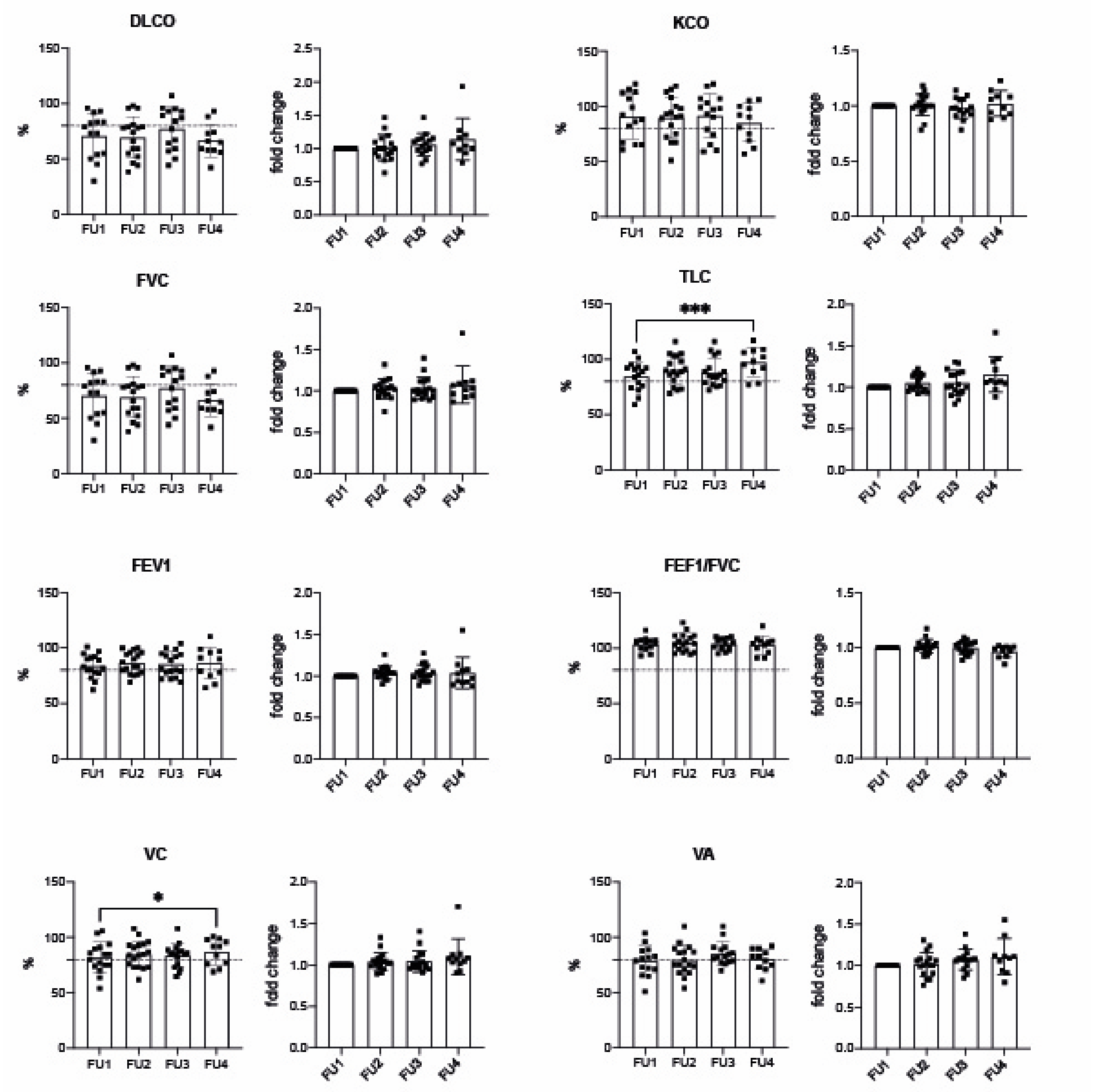

Figure S2. Longitudinal assessment of the pulmonary function. The pulmonary parameters are shown in % of the predicted value (left panels) and fold change of the assessment at the initial follow-up appointment (FU1) (right panels). The dashed line indicates the threshold for abnormal pulmonary function (≤80% of the predicted value). Significance was tested by the one-way-ANOVA with Dunnett's multiple comparison test and is indicated by asterisk (* = $p < 0.05$; *** = $p < 0.001$). Non-significant levels are not labelled. DLCO, lung diffusion capacity for CO; FEV1, forced expiratory volume at the first second of maximal expiration; FU, follow-up appointment; FVC, forced vital capacity; KCO, carbon monoxide transfer rate; TLC, total lung capacity; VA, alveolar volume; VC, vital capacity.

Figure S3

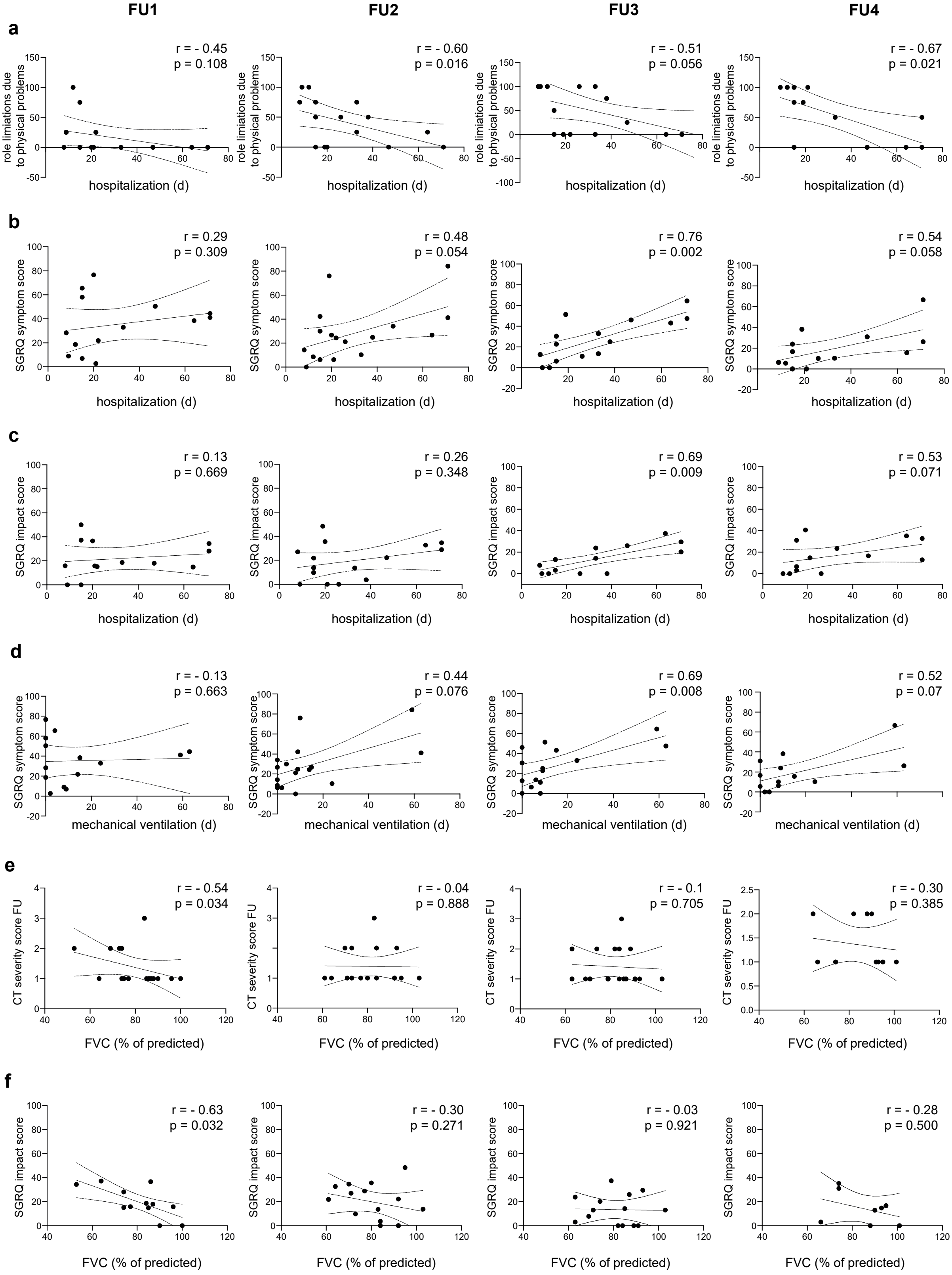

Figure S3. Selected Spearman correlations of clinical data, pulmonary function and HrQoL. (**a**–**f**) The scatter plots show the Spearman correlations with the 95% confidence interval for the FU1-4. The Spearman r and p-values are annotated. CT, computed tomography; d, days; FVC, forced vital capacity; FU, follow-up appointment; SGRQ, St. George's Respiratory Questionnaire

Figure S4

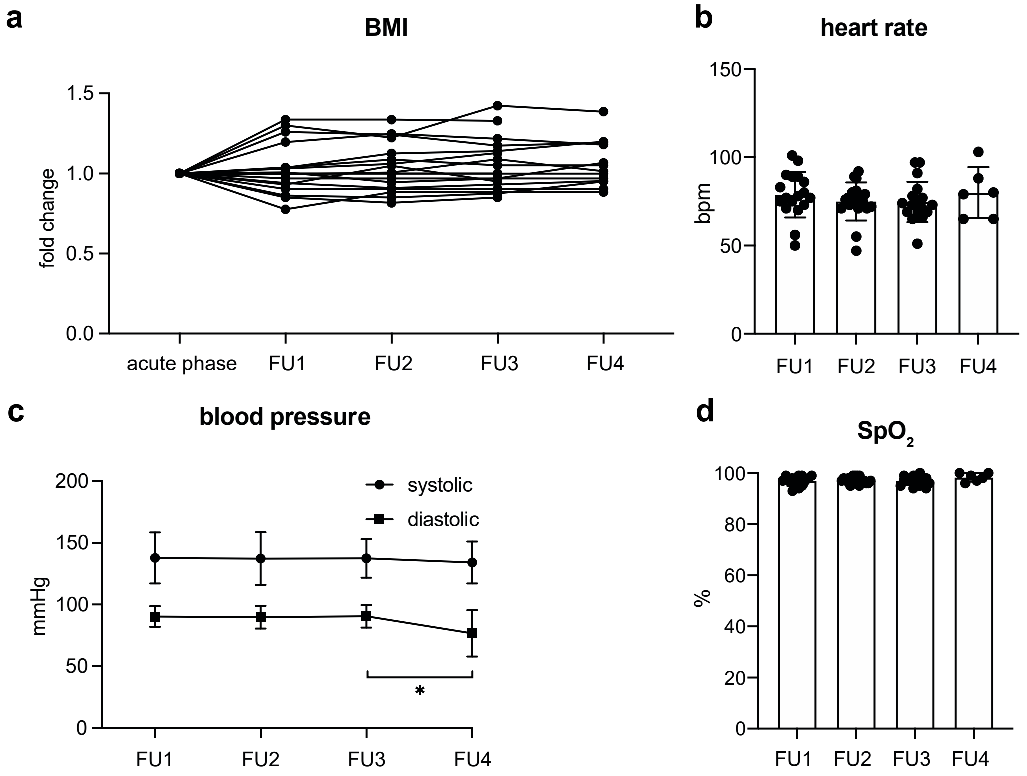

Figure S4. Longitudinal assessment of vital parameters and body mass index. (**a**) The fold change of the body mass index (BMI) at all follow-up appointments (FU) compared to the acute phase is shown. (**b**) The boxplots (mean ± SD) depict all heart rates in beats per minute (bpm) as assessed during the FUs. Dots indicate measurements of individual patients. (**c**) The systolic and diastolic blood pressure (in mmHg) are presented (mean ± SD). (**d**) The peripheral oxygen saturation (SpO_2) of all follow-up appointments (FU) is shown in %. Each data point represents an individual patient. Significance was tested by the one-way-ANOVA with Dunnett's multiple comparison test and is indicated by asterisk (* = $p < 0.05$). Non-significant levels are not labelled.

Figure S5

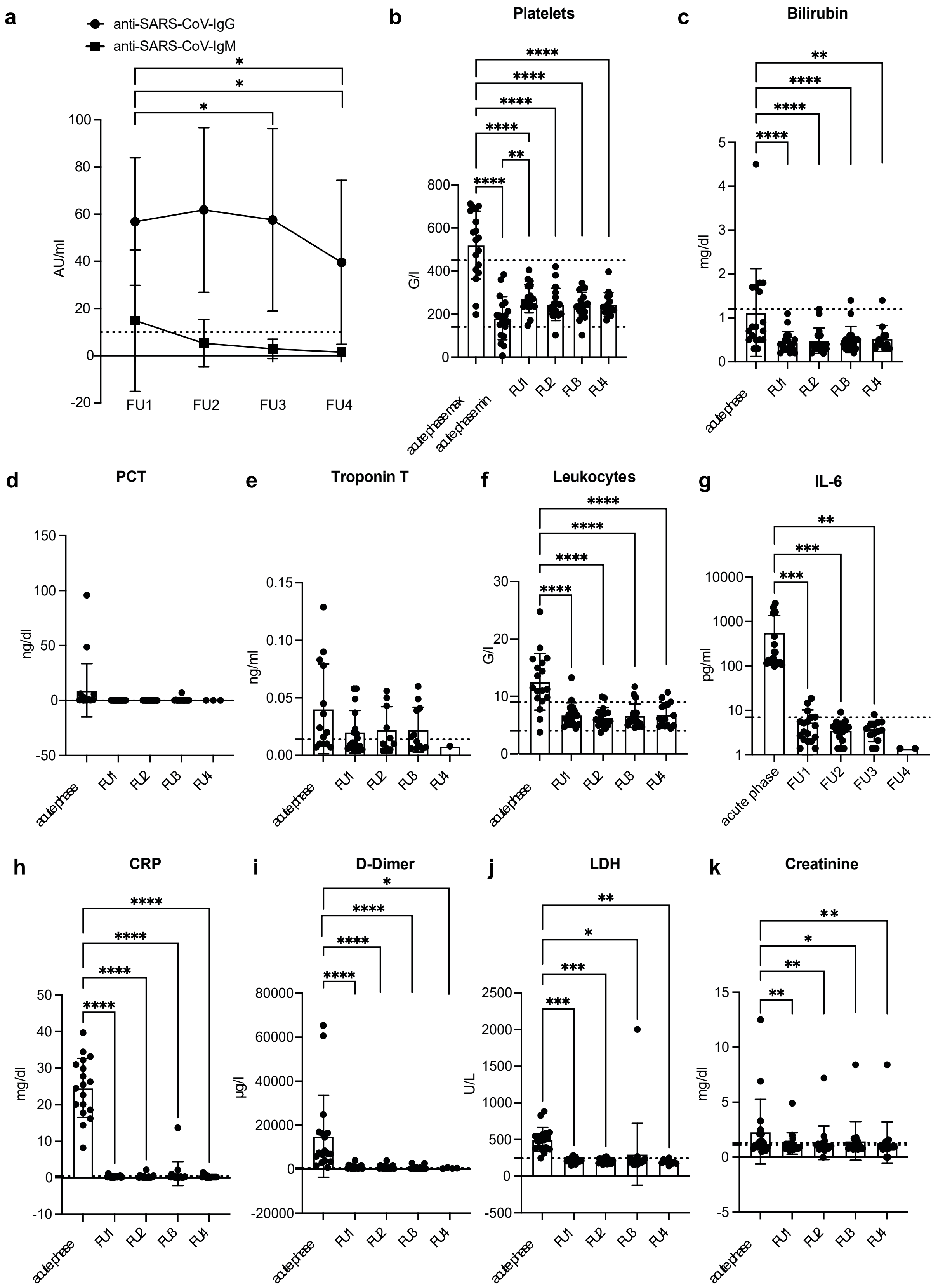

Figure S5. Longitudinal assessment of laboratory parameters and anti-SARS-CoV2 IgG and IgM. (**a**) The anti-SARS-CoV2-IgG and -IgM titers of all follow-up appointments (FU) are presented as mean (±SD). The dashed line indicates the threshold value of 10 IU/ml. Quantitative values were not available for the acute phase. (**b–k**) The boxplots depict the mean (±SD) of the laboratory values. Each data point

represents an individual patient. For (B) the minimum and maximum value of the acute phase is presented. For all other parameters the maximum value is shown. The dashed lines indicate the reference ranges for all parameters. Significance between all FUs and the acute phase was tested by the one-way-ANOVA with Dunnett's multiple comparison test and is indicated by asterisk (* = $p < 0.05$; ** = $p < 0.01$; *** = $p < 0.001$, **** = $p < 0.0001$). Non-significant levels are not labelled. AU, arbitrary unit; CRP, C-reactive protein; IL-6, interleukin-6; LDH, lactate dehydrogenase; PCT, procalcitonin.
